# Confidence of probabilistic predictions modulates the cortical response to pain

**DOI:** 10.1073/pnas.2212252120

**Published:** 2023-01-20

**Authors:** Dounia Mulders, Ben Seymour, André Mouraux, Flavia Mancini

**Affiliations:** ^a^ Computational and Biological Learning Unit, Department of Engineering, University of Cambridge, Cambridge CB2 1PZ, UK; ^b^ Institute of Neuroscience, UCLouvain, 1200 Woluwe-Saint-Lambert, Belgium; ^c^ Institute for Information and Communication Technologies, Electronics and Applied Mathematics, UCLouvain, 1348 Louvain-la-Neuve Belgium; ^d^ Department of Brain and Cognitive Sciences and McGovern Institute, Massachusetts Institute of Technology, MA 02139; ^e^ Wellcome Centre for Integrative Neuroimaging, John Radcliffe Hospital, Headington, Oxford OX3 9DU, UK; ^f^ Center for Information and Neural Networks (CiNet), Osaka 565-0871, Japan

**Keywords:** nociception, confidence, pain, EEG, temporal statistical learning

## Abstract

Pain typically evolves over time, and the brain needs to learn this temporal evolution to predict how pain is likely to change in the future and orient behavior. This process is termed temporal statistical learning (TSL). Recently, it has been shown that TSL for pain sequences can be achieved using optimal Bayesian inference, which is encoded in somatosensory processing regions. Here, we investigate whether the confidence of these probabilistic predictions modulates the EEG response to noxious stimuli, using a TSL task. Confidence measures the uncertainty about the probabilistic prediction, irrespective of its actual outcome. Bayesian models dictate that the confidence about probabilistic predictions should be integrated with incoming inputs and weight learning, such that it modulates the early components of the EEG responses to noxious stimuli, and this should be captured by a negative correlation: when confidence is higher, the early neural responses are smaller as the brain relies more on expectations/predictions and less on sensory inputs (and vice versa). We show that participants were able to predict the sequence transition probabilities using Bayesian inference, with some forgetting. Then, we find that the confidence of these probabilistic predictions was negatively associated with the amplitude of the N2 and P2 components of the vertex potential: the more confident were participants about their predictions, the smaller the vertex potential. These results confirm key predictions of a Bayesian learning model and clarify the functional significance of the early EEG responses to nociceptive stimuli, as being implicated in confidence-weighted statistical learning.

## Significance

The functional significance of EEG responses to pain has long been debated because of their dramatic variability. This study indicates that such variability can be partly related to the confidence of probabilistic predictions emerging from sequences of pain inputs. The confidence of pain predictions is negatively associated with the cortical EEG responses to pain. This indicates that the brain relies less on sensory inputs when confidence is higher and shows us that confidence-weighted statistical learning modulates the cortical response to pain.

In order to survive, animals need to minimize their risk of harm and can do so by learning to predict pain and other body threats. Learning to predict threats is necessary to orient behavior. How does the brain learn to predict pain and aversive states? The majority of previous work has focused on associative learning to predict pain outcomes based on nonpain cues (1–4). Associative learning well describes the prediction of isolated, transient threatening events but is insufficient to characterize learning to predict long-lasting sequences of pain inputs (5), which typically occur in pain conditions (6). When experiencing temporally evolving pain, the brain needs to learn to predict forthcoming pain based on its past history. Recently, we have shown that learning to predict pain sequences can be achieved using optimal Bayesian inference, in the absence of nonpain cues (5). Probabilistic predictions of the frequency of feeling pain are encoded in the human primary and secondary cortex, motor cortex, and right caudate, whereas their precision is encoded in the right superior parietal cortex.

Bayesian inference frameworks make testable hypotheses about the role of confidence in learning and its effect on neural activity. The confidence and error of neural predictions are dissociable measures of uncertainty. Confidence is a measure of the variability of the prediction, irrespective of the outcome of the prediction. In contrast, the prediction error refers to the discrepancy between a prediction and reality. A Bayesian inference account predicts that the confidence of a probabilistic inference 1) weights learning, 2) is integrated with sensory information at early stages of information processing, and 3) is inversely related to sensory cortical responses (i.e., high confidence reduces sensory responses) as the brain relies less on incoming sensory inputs (7, 8). Here, we test these predictions using a TSL task with thermal stimuli and EEG in healthy, human participants.

We focus on the largest wave that can be recorded from EEG in response to transient sensory stimuli: the vertex potential (VP) (9). The VP is typically composed of a biphasic, negative (N2 component) and positive (P2 component) waveform with a characteristic, symmetric scalp distribution with a peak over the vertex (Cz-FCz). The VP can be observed for stimuli in virtually any sensory modality (10), but despite its ubiquity, there is no consensus over its functional significance.

The traditional interpretation is that the VP reflects the intensity of a sensory stimulus (9, 11, 12). A recent study using a pain conditioning paradigm did not find evidence for a modulation of the VP by expectations and prediction errors, suggesting that the VP mostly reflects the sensory processing of a stimulus (13). However, other studies have shown that the amplitude of the VP is modulated by the history and unpredictability of previous stimuli and can be decoupled from perceived intensity (14–18).

**Fig. 1.**
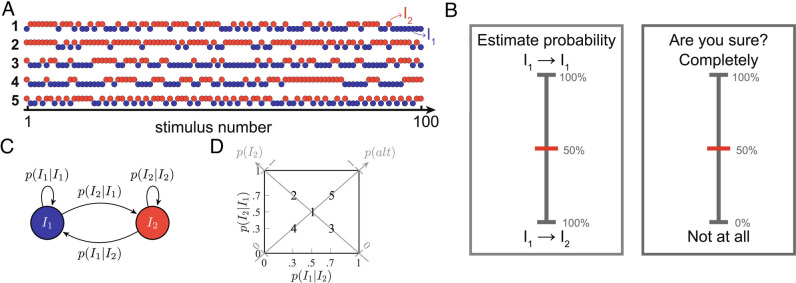
Temporal statistical learning experiment. (*A*) Examples of sequences of stimuli of intensities *I*_1_ and *I*_2_ that are applied to the participants’ forearm. Each sequence has different generative statistics (a majority of *I*_2_ or *I*_1_, more alternations or repetitions, etc.) and the interstimulus interval (ISI) is set to 3 s. (*B*) Behavioral questions asked to the participants every 15 ± 3 stimuli in the sequences to evaluate their stimulus probability estimates and confidence estimates in these predictions. The sequences are paused for a maximum of 8 s per question. (*C*) Markovian generative process of the sequences of stimuli whose intensities are *I*_1_ and *I*_2_. (*D*) Transition probability matrix in which the five generative pairs of transition probabilities (TPs) employed are indicated with bold numbers. One example of a sequence generated with each of these five TPs is shown in (*A*).

The seemingly divergent conclusions of previous studies could stem from the different definitions of stimulus predictability and uncertainty and the lack of a mathematical quantification of these concepts. Here, we use a normative approach to dissect the contributions of temporal predictions, their confidence, and error on the event-related potentials (ERPs) elicited by sequences of somatosensory, thermal stimuli. The stimulus sequences had a probabilistic (Markovian) temporal structure, with underlying statistics that can be learned (Fig. 1) (5).

## Results

**Fig. 2.**
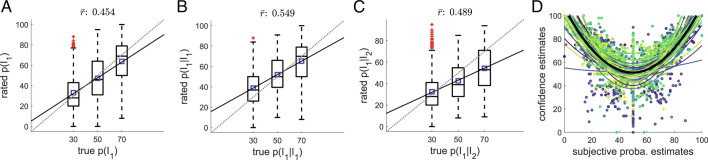
Participants identify the generative sequence statistics. (*A*) True and rated probabilities to receive a stimulus of intensity *I*_1_ are correlated subject-wise (*N* = 31 subjects). The mean correlation across participants is 0.454 (*t*_30_ = 13.603, *P* <  10^−5^, Cohen’s *d* = 2.443), indicating that participants identify the trends within the sequences. The dotted line indicates identity; plain line, linear fit averaged across participants; and blue squares, mean rated probabilities. (*B*) Participants also accurately identify the trends in the transitions from *I*_1_. The grand mean correlation between generative and estimated *p*(*I*_1_|*I*_1_) is 0.549 (*t*_30_ = 14.007, *P* <  10^−5^, Cohen’s *d* = 2.516). (*C*) Similar to (*B*) for the transitions from *I*_2_. The grand mean correlation between generative and estimated *p*(*I*_1_|*I*_2_) is 0.489 (*t*_30_ = 11.585, *P* <  10^−5^, Cohen’s *d* = 2.443). (*D*) Confidence estimates are quadratically related to the probability estimates (mean coefficient of determination of the quadratic fits: *R* ^2^ = 0.47). Plain colored lines indicate individual quadratic fits, and the thick plain black line indicates quadratic fit averaged across participants.

Thirty-one human participants received five different types of probabilistic sequences of thermal stimuli delivered with a contact thermode to the right forearm (Fig. 1*A*). In each sequence, there were two types of stimuli—one stimulus was cool (*I*_1_), and the other was painfully hot (*I*_2_, above the A δ -fiber threshold), to make the task easier and ensure that the participants were able to effortlessly discriminate both intensities. The sequences transitioned between the cool and hot stimuli according to a Markovian process described with two generative transition probabilities (TPs, Fig. 1 *C* and *D*). The participants were asked to try and estimate these TPs. In this task, the primary goal is to clarify how participants perform such inference and how it affects the elicited ERPs, independently of the stimulus intensities used. Occasionally, the sequence was paused and participants were asked to predict the probability of the next stimulus based on the previous stimuli and to report their confidence in these estimates on a numerical rating scale (Fig. 1*B*). Each participant received two sequences of 100 stimuli generated with each of the five distinct TPs indicated in Fig. 1*D* in a randomized order and was informed that the sequence statistics changed across sequences (*M* *e* *t* *h* *o* *d* *s*). On average, along the whole experiment, participants received similar numbers of stimuli from both intensities and rated similar numbers of transitions from both intensities ( *SI Appendix*, Fig. S1). In line with our previous work, participants were able to predict the frequency of the stimulus intensities, as shown by the positive association between generative and rated item frequencies in Fig. 2*A*. Likewise, with a slightly improved accuracy, participants were able to estimate the transition probabilities from one intensity to the other, as indicated in Fig. 2 *B* and *C*. Finally, the confidence estimates were quadratically related to the probability estimates: Confidence estimates tended to increase for more extreme probability estimates, as previously reported for auditory and visual sequences (19).

### Behavioral Modeling.

First, we defined the computational principles underlying the participants’ inference of the sequence statistics. We therefore consider a series of models which are fed with the exact same sequences of binary inputs as the participants. Each of these models constructs predictions about the stimulus probabilities along the sequences and can be compared to the subjective reports to shed light on the mechanisms of pain inference.

We fitted two families of three models to the subjective probability estimates obtained in the statistical learning task. One family of models uses Bayesian inference, whereas the other family uses a heuristic, i.e., a nonprobabilistic delta rule (Rescorla–Wagner model) with a fixed learning rate. The Bayesian models use the confidence of the prediction to weight the update of the representation of the stimulus statistics, whereas delta rule models use a fixed learning rate which is not scaled by uncertainty. In each family, the models differ according to what they predict: the item frequency (IF), the alternation frequency (AF), or the transition probabilities (TPs) of the stimuli.

**Fig. 3.**
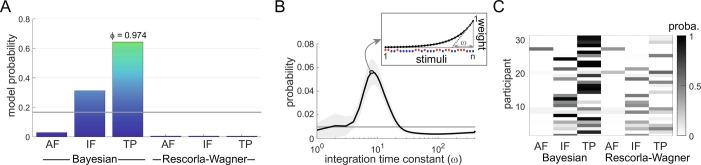
Model comparison. Six different models are considered to explain the subjective reports (*N* = 31 participants): Bayesian learners inferring the alternation frequency (AF), the item frequency (IF) or the transition probabilities (TPs), and delta rule, or Rescorla–Wagner (RW) models, inferring the same sequence statistics (AF, IF, and TP). (*A*) Bayesian model comparison shows that the participants’ reports are best approximated by a Bayesian model learning the TPs (the exceedance probability of this model—i.e., the probability for this model to be more frequent than the others in the population—is *ϕ* = 0.974). Colored bars: model probabilities; horizontal gray line: prior (uniform) probability. (*B*) Bayesian model averaging reveals that the participants’ integration of observations is best approximated with a time constant *ω* of 8 stimuli. Horizontal line: uniform prior probability; shaded area: SEM across participants; plain dot: curve maximum. The inset illustrates the exponentially decreasing weights that are used to count the number of past stimuli when *n* stimuli have been delivered, with a time constant *ω* of 8. (*C*) Individual model probabilities (reflecting the similarity between estimated and modeled probabilities) indicate that most subjective reports are best approximated by the Bayesian model learning the TPs and to a lesser extent by the Bayesian model learning the IFs, but not much by RW models.

At group level, we found that probability estimates were best approximated by a Bayesian model which estimates the transition probabilities (Fig. 3*A*). Given that the sequences were not volatile, we used Bayesian models with fixed update of beliefs and a leaky integration to account for forgetting. We estimated that an integration time constant of approximately 8 stimuli best-approximated behavior (Fig. 3*B*), which corresponds to 24 s and an integration half-life of around 6 stimuli. This provides evidence that statistical learning for nociceptive stimuli uses a Bayesian inference strategy, whereby the update of the representation is weighted by confidence.

A minority of subjects (*n* = 11) favored a simpler Bayesian inference strategy, predicting item frequencies instead of transition probabilities (Fig. 3*C*). This somehow contrasts with our previous study with volatile sequences, in which only a minority of participants could predict the TPs between the stimuli, whereas the majority of participants showed a preference for the simpler strategy of encoding the IF (5). Here, the two models that best approximate the subjective reports and are above the prior uniform probability remain the Bayesian models learning the IF or the TPs, but most participants were able to predict the more complex temporal statistics that are the TPs (Fig. 3*C*). This discrepancy can be explained by the fact that the present task was simplified by the absence of volatility in the generative sequence statistics. Note that frequency can always be derived from transition probabilities (the IF corresponds to the principal diagonal of the TP matrix, Fig. 1*D*), so participants who prefer a transition probability inference strategy should also access the frequency of the stimuli.

**Fig. 4.**
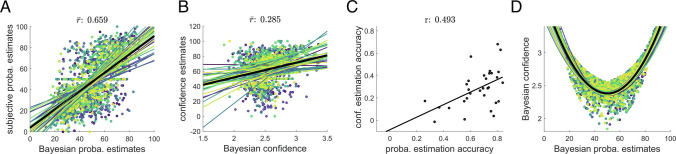
Quality of fit of the best model for the ratings. Subjective estimates of stimulus probability and confidence are highly correlated with Bayes-optimal values obtained from a model learning the TPs with an integration time constant of 8 stimuli (*N* = 31 participants). (*A*) Scatter plot of estimated and modeled stimulus probabilities, with one color per participant. The grand mean correlation is 0.659 (*t*_30_ = 24.398, *P* <  10^−5^, Cohen’s *d* = 4.382). Dotted line: identity; plain colored lines: individual linear fits; thick plain black line: linear fit averaged across participants. (*B*) Scatter plot of estimated and modeled confidence, with the same color code as in (*A*). The grand mean correlation is 0.285 (*t*_30_ = 9.293, *P* <  10^−5^, Cohen’s *d* = 1.669). (*C*) The accuracy of probability and confidence estimates are positively correlated across participants (Pearson correlation: 0.493, *P* = 0.005). Each accuracy was computed as the correlation coefficient between the subjective reports and the corresponding modeled quantities across trials. (*D*) Bayesian confidence is quadratically related to Bayesian probability estimates (mean coefficient of determination of the quadratic fits: *R* ^2^ = 0.59). Plain colored lines: quadratic fits obtained using the sequences of each participant; thick plain black line: quadratic fit averaged across participants’ sequences.

To explore the quality of the fit (i.e., to which extent the winning model is actually close to the participant’s responses), we display the positive correlation between rated and model probability estimates in Fig. 4*A*. Overall, participants’ reports were highly correlated with the model outcomes (grand mean correlation of 0.659, *t*_30_ = 24.4, *P* <  10^−5^). Importantly, the confidence estimates (which were not used to optimize the fit of the model) correlated with the confidence measures deduced from the Bayesian model, Fig. 4*B* (grand mean correlation of 0.285, *t*_30_ = 9.3, *P* <  10^−5^). Bayesian confidence relates to the statistical certainty about the estimated TPs, i.e., to the inverse spread of the posterior distribution over these TPs. The quality of the confidence fit was similar to previous works (20). We then quantified the accuracy of probability and confidence ratings as the correlation coefficients between these estimates and the corresponding model outcomes and found that they were positively correlated across participants (Fig. 4*C*, correlation of 0.493, *P* = 0.005). This indicates that optimizing the model to probability estimates provides a good description of participant’s confidence ratings; it also suggests that confidence and probability estimates are derived from a common cognitive process, in line with previous works (21, 22). Finally, Fig. 4*D* illustrates the quadratic relationship between Bayesian model probability estimates and confidence, similarly to what we observed for the subjective reports (Fig. 2*D*).

### EEG.

**Fig. 5.**
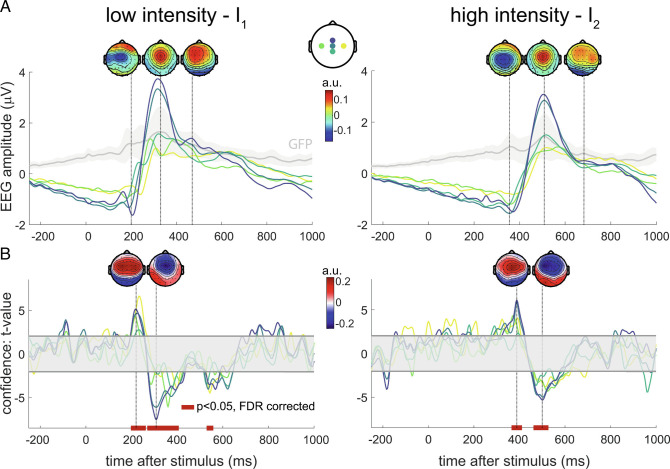
EEG correlates of Bayesian confidence. (*A*) EEG responses averaged over trials, blocks, and participants, for low (*L* *e* *f* *t*) and high (*R* *i* *g* *h* *t*) stimulation intensities. Global field power (GFP) time courses are shown in gray, with shaded SD across participants (*N* = 31). Labels of depicted electrodes, whose positions are shown in the topoplot at the center: C3, Cz, FCz, CPz, and C4. (*B*) Encoding of residual confidence in the EEG responses—*t*-statistics for the regression coefficients associated with model confidence. Confidence is obtained from the model which best explains the participants’ behavior: a Bayesian model learning the TPs with an integration time constant of 8 stimuli. The shaded horizontal areas centered around 0 indicate the nonsignificant regions for *P* <  0.05, two-tailed. Red bars at the bottom of the plots show intervals where the regression coefficients are significantly different from 0 after false discovery rate (FDR) correction of the significance levels. Topographies of the largest effects are indicated.

Sixty-four channel EEG was recorded on all participants while they were exposed to the sequences of thermal stimuli. As expected, the main evoked response consisted in a biphasic waveform—the vertex potential (VP)—which peaked over frontocentral electrodes (9, 23). Fig. 5*A* illustrates the grand-average VPs following cool (*I*_1_) and hot (*I*_2_) stimuli, with scalp topographies of their two main components: the N2 and P2 waves. These two components peaked at 205 ± 17 ms and 318 ± 40 ms after stimulus onset for *I*_1_ and 369 ± 33 ms and 518 ± 42 ms for *I*_2_ (mean ± SD), similar to previous studies using thermal stimulation (12, 24). The VPs in response to both types of stimuli were analyzed separately given their different latencies and thermal qualities. At a single trial level, the earlier N1 wave was not clearly identifiable due to its low signal-to-noise ratio.

Crucially, we investigated whether the confidence and error of the probabilistic inferences modulate the vertex potentials. Using the learning model which best explains the subjective reports (a Bayesian model learning the TPs with an integration time constant of 8 stimuli), we regressed the single-trial EEG signals on two distinct inferential quantities: the model residual confidence and Bayesian prediction error (BPE). Confidence is defined as the log precision of the posterior distribution over the latent parameter and is therefore inversely proportional to the posterior variance—confidence gets higher when the variance gets smaller Eq. **7**. The residual confidence is obtained from the confidence by regressing out the predicted probability, its square, and its logarithm to ensure that these quantities do not drive the effects of modeled confidence (*M* *e* *t* *h* *o* *d* *s* and Eq. **14**) (20). Besides, BPE corresponds to the difference between the received intensity and its predicted probability in the model Eq. **8**. For each participant, we included these two regressors in linear regressions at each time point from −0.5 to 1 s around stimulus onset and at central electrodes of interest (C3, Cz, FCz, CPz, and C4). To make sure that BPE and confidence were not collinear, confidence was regressed on BPE subject-wise, leading to average variance inflation factors (VIFs) of 1 and 1 for *I*_1_ and *I*_2_ respectively, (regression *R* ^2^ <  10^−5^). Two variables are typically considered to be highly collinear when their VIF is above 5 (25).

**Fig. 6.**
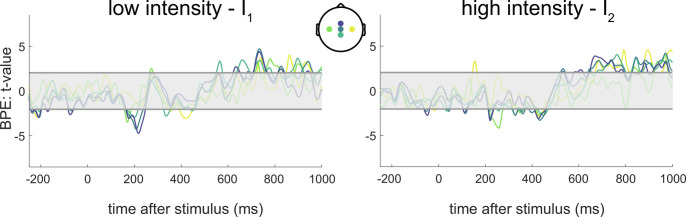
EEG correlates of Bayesian prediction error (BPE). Encoding of BPE in the EEG responses, similar to Fig. 5*B*—*t*-statistics for the regression coefficients associated with BPE. BPE is obtained from the model which best explains the participants’ behavior: a Bayesian model learning the TPs with a time constant of 8 stimuli. The shaded horizontal areas centered around 0 indicate the nonsignificant regions for *P* <  0.05, two-tailed. No time interval was deemed significant after false discovery rate (FDR) correction of the significance levels.

Grand averages of the *t*-statistics obtained from *t*-tests against 0 for the regression coefficients are shown in Figs. 5*B* and 6. First, we found a clear modulation of the VP by residual confidence for both intensities (Fig. 5*B*). The sign of these modulations is opposite to the VP, meaning that the larger the model confidence, the smaller the N2 and P2 components.

*SI Appendix* analyses show that using confidence instead of residual confidence leads to comparable observations ( *SI Appendix*, Fig. S2, even though the VIFs are slightly larger in this case). Considering the best fitting model for each individual participant (model learning IF, AF, or TPs) also leaves these outcomes unchanged ( *SI Appendix*, Fig. S3). If the Bayesian model learning the IF instead of the TPs is considered (second best model fitted to the behavioral reports), results are also similar ( *SI Appendix*, Fig. S4).

Finally, we found no statistical evidence for a modulation of the BPE on the EEG potentials, after correcting for the false discovery rate (Fig. 6). However, the prediction error derived from a Bayesian model learning the IF instead of the TPs significantly modulates late EEG waves ( *SI Appendix*, Fig. S4). The IF model typically leads to more confident predictions than the TP model because it is simply inferring one parameter (the frequency) rather than two transition probabilities. However, the IF model predictions are more likely to be “wrong” than the TP model predictions because the sequences of stimuli were generated using TPs rather than only IFs. Bigger BPEs should yield stronger modulations of the late EEG waves, according to a hierarchical Bayesian inference framework. This is what we find, i.e., the IF BPE modulates more consistently late cortical responses than the TP BPE.

## Discussion

The brain needs to learn to predict forthcoming nociceptive stimuli in order to minimize potential harm. When pain persists over time, the brain needs to extract and learn structure or patterns from streams of sensory inputs without relying on explicit feedback or associated cues (26, 27). Using a statistical learning task in conjunction with EEG, we provide evidence in support of the view that the human brain uses confidence-weighted Bayesian inference to learn to predict future pain levels (28) and that confidence modulates the cortical response to pain (29–32). First, we found that subjective probability estimates of thermal sensations and the associated confidence reports are well approximated by a Bayesian inference model. The best fitting model learns the transition probabilities within the sequences and accounts for participants’ forgetting by integrating past observations with a time constant of 8 stimuli (24 s). At the opposite of non-Bayesian models, this winning model indicates that the effect of prior expectations is weighted by confidence to predict forthcoming nociceptive inputs (3, 5, 33). Second, the modeled confidence was negatively associated with the amplitude of the vertex potential (VP): The higher the participants’ confidence in the intensity prediction, the smaller the VP. Prediction errors (PEs), measuring the discrepancy between the expected stimulus and the one which was received, were only weakly associated with increases in later EEG responses. These findings were predicted by our hierarchical Bayesian processing hypothesis: High confidence reduces the cortical response to thermal stimuli because the brain relies less on incoming sensory information and more on prior information, to generate an inference.

The notion of confidence corresponds to a “feeling-of-knowing” about some variables in an uncertain environment (21). It is important to note that this notion is employed in two kinds of situations, leading to different computational definitions of confidence. First, confidence in a discrete variable that is learned can be quantified by the probability for this variable to take a given value; it corresponds to the so-called choice or decision confidence (34–38). Second, confidence in the value of a continuous variable instead relates to the spread (often quantified by the SD) of the estimated posterior distribution of this variable (21, 38, 39). For instance, in a TSL task like in this work, the confidence in the next stimulus intensity corresponds to the estimated probability to receive this intensity, while the confidence in the sequence statistic that is learned (AF, IF, or TP) is related to its estimated SD. As a consequence, decision confidence—which has been the object of numerous publications about choice and decision-making—should not be confounded with the inferential confidence studied here. For the EEG analysis presented in Fig. 5, the estimated probability of each intensity has been regressed out to obtain the residual confidence, which is neither linearly nor quadratically related to decision confidence.

Statistical models of sensory perception predict that inferential confidence should serve as a weighting factor increasing the effect of prior beliefs on perception (7, 32, 33). In the pain field, a few works have studied this principle: From a behavioral viewpoint, confidence indeed modulates pain perception by weighting the effect of expectations (29, 30, 32). While it is clear that individuals are able to provide metacognitive judgments about pain to some extent (40), some studies suggested that humans have a less accurate sense of confidence in the sensory discrimination of pain compared to other sensory modalities (41). This contrasts with our finding that inferential confidence is correlated with the Bayesian model confidence, suggesting that it is derived from a near-optimal inference process. However, our study did not focus on fitting different metacognitive models to the subjective reports, and the match between modeled and estimated confidences is not perfect—this indicates that there might be other contributions to the actual confidence estimates, including metacognitive bias or variability (41, 42).

Regarding the effects of confidence on brain response dynamics, in a hierarchical Bayesian framework, we would expect to see early modulations of EEG responses by confidence, such that increased confidence would lead to a reduction of these responses (8, 32). The few existing studies that looked at confidence effects on EEG/MEG signals are consistent with this view (31, 32) and suggest that confidence for pain is encoded in the somatosensory cortex (28), but they have not tested its key predictions on the main EEG responses to pain. Here, we show that confidence in statistical inference has a negative association with an early cortical response to nociceptive stimuli, i.e., the VP. The functional significance of the VP has been debated for decades. Traditionally, it was thought that the VP reflects the sensory processing of a stimulus, and it is indeed often used in clinical neurophysiology as a marker of sensory function (9, 11, 12). Using nociceptive stimuli, the VP has been associated with subjective pain intensity, and, as such, it could be influenced by perceptual and attentional mechanisms (43, 44). Other works have shown that the VP is more likely to encode the differential intensity of a stimulus (with respect to baseline) rather than its absolute intensity (45). Besides, several studies have emphasized that the VP amplitude is not only affected by stimulus intensity and the recent history of stimulation but also by the unpredictability, novelty, and saliency of each stimulus (15, 31, 46, 47). For instance, just repeating the same stimulus a few times induces a dramatic habituation of the VP, despite the fact that perception remains stable and peripheral habituation can largely be ruled out (e.g., because a new skin spot has been stimulated after each stimulus) (18, 46). Still, a more recent study using a cued pain paradigm suggested that the VP is mostly associated with the sensory processing of a stimulus, without being affected by expectations and PEs (13). These different interpretations can result from the lack of a computational quantification of the pain learning process on a trial basis that would enable fitting individual learning models to each participant (41, 48). Indeed, the aforementioned works did not have estimates of uncertainty or confidence at an individual level because they relied on axiomatic approaches and/or cue-based paradigms. Here, we introduce a computational approach which quantifies nociceptive inference trial by trial, enabling the direct correlation of information processing quantities to their brain encoders instead of limiting the contextual information to binary intensities or discrete stimulus and cues categories.

Another component of the statistical learning process is the generation of prediction errors (PEs), measuring the difference between what is predicted (based on previous experiences) and what is actually received. PEs (or surprise) signals are expected to modulate some brain responses regardless of the sensory modality (49), though it is likely that the neural implementation of these effects have some stimulus specificity (50). Here, we did not find significant evidence for an effect of PE on the VP, although there was a weak modulation of late-onset EEG responses. In different paradigms, using shorter sequences of stimuli, PEs can account for shorter time-scale habituation (4, 45). This is not incompatible with our findings: in short and/or cued sequences, PEs tend to be large, and this is likely to lead to a stronger cortical modulation, as dictated by Bayesian inference.

To conclude, we have shown that subjective probability reports about nociceptive intensity are well approximated by a Bayesian model learning the transition probabilities between high and low-intensity stimuli. The Bayesian model’s confidence was correlated with the participants’ reported confidence levels. Importantly, inferential confidence was negatively correlated with the VP— the higher the confidence, the smaller the VP. This indicates that the VP is modulated by confidence-weighted statistical learning of sequences of nociceptive inputs and is consistent with the predictions of a hierarchical Bayesian inference framework. Given that some pathological pain conditions have been associated with altered learning and predictive capabilities (51–54), future works could assess how confidence representations are modified in these patients, opening the path to promising translational studies.

## Materials and Methods

### Participants.

Thirty-six healthy participants (19 females) were recruited for the experiment, 32 of them being right handed. The study was approved by the local ethics committee (Comité d’Ethique Hospitalo-Facultaire de l’Université catholique de Louvain, B403201316436). All participants gave written informed consent and received financial compensation. Five participants were excluded from the analyses for the following reasons:

- participant #1 was a pilot subject and was excluded because different stimulation parameters were used for her session than the experimental group (500-ms instead of 250-ms stimuli and a lower *I*_2_),
- participants #15 and #33 fell asleep during the experiment, and their data collection was therefore stopped,
- participant #11 made one mistake during a precheck stimulus discrimination session (*P* *r* *o* *c* *e* *d* *u* *r* *e*) and the experiment was terminated,
- and participant #28 made two errors during the postcheck stimulus discrimination session (*P* *r* *o* *c* *e* *d* *u* *r* *e*) and was hence excluded.

The procedure used to check discrimination is described below. After this exclusion, there were 31 subjects (16 females) left, aged 18 to 30 y.

### Experiments.

The task aims to assess temporal statistical learning (TSL) using sequences of nociceptive stimuli of two distinct intensities—*I*_1_ and *I*_2_. The core principle is that as participants are exposed to a stream of stimuli, they are able to track the sequence statistics to some extent. Indeed, as the sequence goes, one collects evidence of whether the sequence contains more *I*_1_, more *I*_2_, systematically more *I*_1_ following *I*_1_ or *I*_2_, etc. In our experiment, we aim to understand how these learning mechanisms are implemented.

#### Stimuli and Generative Model.

The stimuli were 250-ms-long thermal pulses, applied to the participant’s right volar forearm with a contact thermode (QST Lab, Strasbourg, France, active stimulation surface: 120 mm^2^, heating and cooling ramps of 300 ^°^/s, no active baseline temperature). To ease the task and ensure that the participants were able to easily identify the stimulus intensities along all the tested sequences, the intensity *I*_1_ was chosen to be nonpainful and cool, while the intensity *I*_2_ was selected to be painful and above the individual A δ fiber threshold while being bearable. The temperatures employed were therefore *I*_1_ = 15 ^°^C and *I*_2_ = 58 ^°^C, up to modifications based on individual thresholds and/or discrimination capabilities, as detailed below. The intensity *I*_2_ was described as painful and pricking by all participants.

The stimulus intensity $y_{n}\in \left\{I_{1},I_{2}\right\}$ at each time step *n* along a given sequence is uniquely generated according to a two-state Markovian process such that

-
$p\left(y_{1}=l_{1}\right)=\frac{p\left(l_{1}|l_{2}\right)}{p\left(l_{1}|l_{2}\right)+p\left(l_{2}|l_{1}\right)}.$

-
$p\left(y_{n}|y_{1:n-1}\right)=p\left(y_{n}|y_{n-1}\right).$

Each sequence is therefore characterized by its generative transition probabilities (TPs, (*p*(*I*_1_|*I*_2_),*p*(*I*_2_|*I*_1_))), i.e., the probabilities of either intensity given the previous stimulus intensity.

#### Procedure.

Each participant underwent the following steps: 1) A δ fibers threshold estimation through a staircase procedure using reaction times, 2) one precheck block to assess the discrimination of the two stimulus intensities, 3) one training block, 4) 10 testing blocks, and 5) one postcheck block to reassess the discrimination of the two stimulus intensities at the end of the experiment. The total duration of the experiment was approximately 3 h.

#### A δ fibers threshold estimation.

The threshold for activating A δ fibers was determined with an adaptive staircase procedure using reaction times (RTs) as described in ref. (55). A 250-ms heat stimulus was assumed to activate A δ fibers when the perception RT was ≤ 650 ms. Starting with a 45 ^°^C-stimulus, temperature was increased until the RT became shorter than 650 ms, which led to decrease the next stimulus temperature. The successive absolute temperature differences were in {5, 2, 1, 0.1} ^°^C, decreasing after each detection change (RT shorter vs. longer than 650 ms). The threshold was defined as the mean of 4 stimulation temperatures, which led to three consecutive changes of RT shorter vs. longer than 650 ms. This led to thresholds of 52.7 ^°^C (±5.1) on average (±SD).

#### Check blocks.

During each precheck and postcheck block, the participant received a random sequence of 15 stimuli with intensities *I*_1_ and *I*_2_ fully random TPs of (0.5, 0.5) and self-paced interstimulus intervals (ISIs). After each stimulus, the participant was asked to report the stimulus identity (cool or hot), and the thermode was displaced before delivering the next stimulus. If there were more than 1 mistake in a precheck block, hesitations about the stimulus identity, or if *I*_2_ were unbearable, the stimulus intensities were adjusted accordingly. This led to increase *I*_1_ to 20 ^°^C for four participants, decrease *I*_2_ to 57 ^°^C for 11 participants, and exclude one participant who could not clearly identify the stimuli. After these adjustments, if a participant made any further errors in the precheck session, the experiment was terminated (this happened once). If there were more than 1 mistake in a postcheck block during the last sequences, the participant was excluded from the analyses (this happened once). We did not allow a single error in the precheck or postcheck sessions because the temperature difference between the two stimuli was very large (on average 43 ^°^C); we reasoned that any error in the prechecked or postchecked sessions was likely to be due to inattention in a healthy subject. *SI Appendix*, Table S1 indicates the outcomes of the check blocks, the temperatures used, and the exclusion reasons for all subjects who were recruited.

#### Training and testing blocks.

During a training or testing block, the participant was exposed to one sequence of stimuli whose intensities were generated based on fixed TPs. The thermode was displaced on the forearm between successive stimuli to avoid trial-to-trial habituation and sensitization which could prevent the participant from easily distinguishing the two intensities and/or suppress the A δ response before the sequence end. The within-sequence ISI was set to 3 s to leave enough time to slightly displace the thermode while avoiding a confound between tactile and thermal components in the recorded responses. The experimenter was equipped with an earpiece through which a sound signaled the end of each stimulus +0.3 s, as an instruction to initiate the displacement. Every 15 ± 3 stimuli, the sequence was paused to probe the participant’s inference of the sequence TPs—the participant was asked to 1) estimate the probability of the next stimulus intensity and then 2) rate their confidence in this estimate, Fig. 1*B*. The scales were displayed on a computer screen in front of the participant and numerical ratings were collected based on keyboard inputs. A time limit of 8 s was set to answer each question to avoid too long breaks within the sequences, which could affect learning (56).

The **training block** consisted of one sequence of 50 stimuli generated with TPs (0.7, 0.4) and enabled the participants to understand the generative process and familiarize themselves with the task. Subjects received feedback at the end of this sequence on the correctness of their rating trend.

In each of the **10 testing blocks**, the participant received one sequence of 100 stimuli. The first and last five sequences were generated with the five different TPs indicated with numbers in Fig. 1*D*: (0.5, 0.5), (0.3, 0.7), (0.7, 0.3), (0.3, 0.3), and (0.7, 0.7). The order of the blocks was randomized across participants, and variable breaks were allowed between sequences.

Behavioral data were analyzed with Matlab R2019b (The MathWorks), and Cohen’s *d* is reported as effect size for each t -test.

### Learning Models.

The generative parameters of the sequence can be continuously estimated based on the stimuli received, leading to predictions about the forthcoming stimulus. To understand how participants perform this inference task, different models performing the same task were fitted to the subjective probability estimates and compared.

Two families of learning models were considered to explain the sequence statistics inference: a Bayesian learner and a non-Bayesian Reinforcement Learning (RL) model which is called the delta rule or Rescorla–Wagner (RW) model (19, 33, 57).

#### Bayesian model.

A Bayesian model estimates the posterior distribution of a latent parameter *θ* given the sequence of observed stimuli $y_{1:n}$ at each time step *n* using Bayes’ rule (19). Each model *M* estimates specific sequence parameters: either the item frequency (IF) or the alternation frequency (AF) or the transition probabilities (TPs). Given a model *M*, the parameter posterior is obtained by combining the parameter prior and the likelihood of past observations:

[1] $$\begin{array}{cc} p(\theta |y_{1:n},M) & \propto p\left(y_{1:n}|\theta ,M\right)\cdot p\left(\theta |M\right). \end{array}$$

We use a uniform (conjugate) prior distribution over the parameter values, i.e., *p*(*θ*|*M*)∼Beta(*θ*|1, 1), which enables deriving analytical solutions for the posterior. Using the Markovian assumption $p\left(y_{n+1}|y_{1:n},\theta \right)=p\left(y_{n+1}|y_{n},\theta \right)$ , the likelihood can be decomposed as

[2] $$\begin{array}{cc} p(y_{1:n}|\theta ,M) & =p\left(y_{n}|y_{n-1},\theta ,M\right)\cdot \ldots \cdot p\left(y_{3}|y_{2},\theta ,M\right)\cdot \\ & p\left(y_{2}|y_{1},\theta ,M\right)\cdot p\left(y_{1}|\theta ,M\right). \end{array}$$

This likelihood and thereby the posterior can be further simplified depending on the model *M* as shown below.

1. **IF learning**. With this model, the inferred parameter is the probability to receive a stimulus of intensity *I*_1_: *θ* = *p*(*I*_1_):=*θ*_*I*_1__. The posterior is therefore
[3] $$\begin{array}{cc} p(\theta_{I_{1}}|y_{1:n},M) & ∼\;\text{Beta(}\theta_{I_{1}}|N_{1}+1,N_{2}+1\text{)}, \end{array}$$
  where *N*_1_ and *N*_2_ are the numbers of stimuli of intensity *I*_1_ and *I*_2_ respectively within $y_{1:n}$ .
2. **AF learning**. The inferred parameter is the probability of intensity alternation, i.e., the probability to switch from *I*_1_ to *I*_2_ or vice versa within the sequence: *θ* = *p*(alt.): = *θ*_alt._. The posterior distribution reads
[4] $$\begin{array}{cc} p(\theta_{\;\text{alt.}}|y_{1:n},M) & ∼\;\text{Beta(}\theta_{\;\text{alt.}}|N_{a}+1,N_{r}+1\text{)}, \end{array}$$
  with *N*_*a*_ and *N*_*r*_ the number of alternations and repetitions of stimulus intensities within $y_{1:n}$ .
3. **TPs learning**. The inferred parameter is now two-dimensional and corresponds to the transition probabilities of the sequence of stimuli: *θ* := (*θ*_*I*_1_|*I*_2__, *θ*_*I*_2_|*I*_1__), which leads to the posterior
[5] $$\begin{array}{cc} p(\theta |y_{1:n},M) & ∼\;\text{Beta(}\theta_{I_{1}|I_{2}}|N_{1|2}+1,N_{2|2}+1\text{)}\cdot \\ & \;\text{Beta(}\theta_{I_{2}|I_{1}}|N_{2|1}+1,N_{1|1}+1\text{)}, \end{array}$$
  where *N*_*j*|*k*_ is the number of transitions from *I*_*j*_ to *I*_*k*_ counted within $y_{1:n}$ .

To account for limited memory constraints during inference and an unknown timescale of integration, a leaky integration of observations is considered (19). All the models are endowed with a free parameter *ω* ∈ [1, ∞]—the integration time constant—and the *k*th last observation counted (being it an item, an alternation, or a transition depending on the model considered) is weighted according to an exponential decay by a factor exp^−*k*/*ω*^.

For all Bayesian models, some outcomes of interest can be deduced from the posterior at each position *n* within the sequence, when the observations *y*_1 : *n*_ have been received:

- **The probability of the next stimulus** is the mean of the posterior distribution:
[6] $$\begin{array}{cc} p(y_{n+1}|y_{1:n},M) & =\int_{0}^{1}p\left(y_{n+1},\theta |y_{1:n},M\right)d\theta \\ & =\int_{0}^{1}p\left(y_{n+1}|\theta ,y_{n},M\right)\cdot p\left(\theta |y_{1:n},M\right)d\theta . \end{array}$$
- **The confidence in the learned parameter** relates to the precision (inverse variance, *π* := 1/*σ* ^2^) of the posterior (33, 38):
[7] $$\begin{array}{cc} c_{n} & =-\log\left(\sigma \left(p\left(\theta |y_{1:n},M\right)\right)\right)=0.5\cdot \log\left(\pi \left(p\left(\theta |y_{1:n},M\right)\right)\right). \end{array}$$
  The confidence quantifies the certainty in the estimated continuous variable and is typically expressed in log space so that the SD and variance are proportional.
- **The prediction error** is defined like in a Bayesian predictive coding framework (58, 59) as
[8] $$\begin{array}{cc} e_{n} & =1-p(y_{n}|y_{1:n-1},M). \end{array}$$
  It can be noted that, likewise, the Shannon surprise (33) elicited by the last stimulus also quantifies the discrepancy between the intensity that was expected and the one that is received (*y*_*n*_), in a log space: *s*_*n*_ = −log(*p*(*y*_*n*_|*y*_1 : *n* − 1_, *M*)). Examples of posterior distributions and their mean before (in gray) and after (in black) receiving a stimulus within the sequence are shown in *SI Appendix*, Fig. S5, illustrating the concepts of confidence and prediction errors.

To assess the extent to which these models and their parameter (the integration time constant) are identifiable in our experiment, parameter and model recovery analyses can be found in *SI Appendix*, Fig. S6.

#### Rescorla–Wagner, or delta rule, models.

The delta rule model, or Rescorla–Wagner (RW) model (57, 60), is compared to the Bayesian model. While the latter weights the posterior updates by confidence (33), the delta rule uses a constant and nonstatistical weighting of incoming observations to estimate the latent parameter. The inferred parameter *θ* (IF, AF, or TPs) is initiated at 0.5 and is seen as a state value *V* in the RW models, as detailed in what follows.

1. **IF learning**. The state value corresponds to the estimated probability to receive a stimulus of intensity *I*_1_: $V_{n}:={\hat{\theta }}_{I_{1},n}$ .
  At each step *n* in the sequence, the state is updated as
[9] $$\begin{array}{cc} V_{n} & =V_{n-1}+\alpha \cdot \left(R_{n}-V_{n-1}\right),\\ & \;\text{where}\;R_{n}=1\;\mathrm{if}\;y_{n}=I_{1}\;\mathrm{and}\;R_{n}=0\;\mathrm{if}\;y_{n}=I_{2}, \end{array}$$
  and with the learning rate *α* ∈ ]0, 1[ being a free model parameter.
2. **AF learning**. The state value corresponds to the estimated probability of an alternation within the sequence: $V_{n}:={\hat{\theta }}_{\;\text{alt.},n}$ .
  The state is updated as
[10] $$\begin{array}{cc} V_{n} & =V_{n-1}+\alpha \cdot \left(R_{n}-V_{n-1}\right),\\ & \;\text{where}\;R_{n}=0\;\mathrm{if}\;y_{n}=y_{n-1}\;\mathrm{and}\;R_{n}=1\;\;\text{otherwise}. \end{array}$$
3. **TPs learning**. The state value is two-dimensional and corresponds to the estimated transition probabilities: $V_{1,n}:={\hat{\theta }}_{I_{1}|I_{1},n}$ , $V_{2,n}:={\hat{\theta }}_{I_{1}|I_{2},n}$ .
  The state is updated as
[11] $$\left\{\begin{array}{l} V_{i,n}=V_{i,n-1}+\alpha \cdot (R_{n}-V_{i,n-1}),\;\text{if}y_{n-1}=I_{i},\\ \;\;\;\;\;\;\;\text{with}R_{n}=1\text{if}y_{n}=I_{1}\text{and}R_{n}=0\text{if}y_{n}=I_{2},\\ V_{i,n}=V_{i,n-1}\;\text{if}y_{n-1}\neq I_{i}. \end{array}\right.$$

### Model Fitting.

To determine to which extent each model accounts for the subjective reports, we quantify the relationship between subjective and model probability estimates by linearly regressing the subjective reports on the modeled estimates for each participant and model. Across trials indexed by *n*, the probability report *x*_*n*_ is hence regressed on the model probability of *I*_1_ *p*_*n*_^*M*_*i*_, *ω*_*i*_^ deduced from each model *M*_*i*_ with free parameter *ω*_*i*_ as described above (Bayesian and RW models learning the IF, AF, or TPs, with integration time constant or learning rate as a free parameter) as:

[12] $$\begin{array}{cc} x_{n} & =\beta_{0}+\beta_{1}\cdot p_{n}^{M_{i},\omega_{i}}+\epsilon , \end{array}$$

where *β* are the regression coefficients, estimated by OLS, and *ϵ* the residuals.

The quality of this fit is quantified by the model evidence (or marginal likelihood) *p*(*x*|*M*_*i*_), which is estimated with the Bayesian information criterion (BIC) as:

[13] $$\begin{array}{cc} p(x|M_{i}) & \approx \exp(\frac{-BIC}{2}), \end{array}$$

with BIC =*N* ⋅ log(*σ*_*e*_^2^)+*q* ⋅ log(*N*), the mean squared error (MSE) of the regression $\sigma_{e}^{2}=\min_{\omega_{i}}\frac{1}{N}\sum_{n=1}^{N}{\left(x_{n}-{\hat{x}}_{n}^{M_{i},\omega_{i}}\right)}^{2}$ , *N* the number of observations and *q* the number of parameters (here there are two regression coefficients and one model-free parameter). When comparing models with the same number of parameters, minimizing the BIC amounts to minimizing the MSE. We considered 99 possible learning rates for the RW models in the range from 0.005 to 0.95, and 103 integration time constants for the Bayesian models from 1 to 400 plus infinity (i.e., a perfect integrator).

Individual, subject-wise, model probabilities were obtained by normalizing the model evidence estimated with the BIC as in Eq. **13**.

### Model Comparison.

The model with the largest model evidence (or lowest BIC) was considered to be the best fit for the ratings. To compare the six models *M*_*i*_ described above, we conducted a Bayesian model comparison as implemented in the VBA toolbox (61) and adopted a random-effect approach, assuming that the optimal model can differ across participants. The analysis yielded the expected probability of each model *M*_*i*_ and the probability for *M*_*i*_ to be more frequent than all the other models in the population, which is called the “exceedance probability” and is denoted by *ϕ*.

The model-free parameter which approximated the subjective reports best on average was determined through Bayesian model averaging (49) for the Bayesian and RW models separately by estimating *p*(*ω*|*x*) ∝ ∑_*i*_ *p* (*x*|*M*_*i*_, *ω*) ≈ ∑_*i*_exp(−BIC(*M*_*i*_, *ω*)/2).

### Electrophysiological Recordings.

EEG was recorded during the whole experiment using 64 Ag–AgCl electrodes placed on the scalp according to the international 10/10 system (WaveGuard 64-channel cap, Advanced Neuro Technologies) and with an average reference. The synchronization of the stimuli, triggers on the EEG, and behavioral questions was performed with the Data Acquisition Toolbox and Psychtoolbox running on Matlab. Electrode impedances were kept below 10 kΩ. Eye movements were recorded using a pair of surface electrodes placed above and on the right side of the right eye, and one electrocardiogram (EKG) lead was recorded with two surface electrodes, one below the right clavicle near the shoulder and the other on the last left rib. Signals were amplified and digitized at 1,000 Hz. Participants were asked to move as little as possible and keep their gaze fixed on the computer screen in front of them, which displayed a fixation cross and occasional behavioral questions (*E* *x* *p* *e* *r* *i* *m* *e* *n* *t* *s*).

#### Preprocessing.

The EEG recordings were analyzed using Matlab R2019b (The MathWorks). First, the following preprocessing steps were conducted using Letswave 6 (http://letswave.org) (62): high-pass filtering above 0.5 Hz with a 4th order zero-phase Butterworth filter, 50-Hz bandpass notch filtering, downsampling to 500 Hz, segmentation of trials from −1 to +1.5 s relative to stimulus onsets, baseline mean correction, and rejection of stereotyped artifacts using an independent component analysis (ICA) decomposition (63). Then, using Matlab, epochs were low-pass filtered below 30 Hz, and trials with amplitudes reaching 80 μV were rejected, leading to keep 491 ± 17.3 and 490.2 ± 16.27 (grand mean ± SD) stimuli of intensities *I*_1_ and *I*_2_. We also extracted gamma-band oscillations, a typical EEG correlate of pain perception (54, 64); details and results are reported in *SI Appendix* ; ( *SI Appendix*, Fig. S7).

#### Linear regressions.

We sought to determine whether and how the vertex potential (VP) reflects the behavioral outcomes observed during TSL. The model which best approximated the participants’ behavior was considered (Bayesian model learning the TPs with a time constant *ω* = 8), and the VP was regressed on its key inferential outcomes. Two regressors were included in the analysis: the prediction error, Eq. **8**, known to affect sensory responses (4, 49), and the confidence in the estimates, which weights learning in a Bayesian framework (33) Eq. **7**.

To ensure that the effects of confidence on EEG signals were not driven by confounding factors related to the prediction itself ( $p\left(I_{1}|y_{1:n},M_{i},\omega_{i}\right):=p_{n}$ ) (20), we first computed the residual confidence $c_{n}^{r}$ from the confidence *c*_*n*_ by regressing out the predicted probability, its logarithm, and its square as:

[14] $$\begin{array}{c} c_{n}=\beta_{0,k}^{r}+\beta_{1}^{r}\cdot p_{n}+\beta_{2}^{r}\cdot p_{n}^{2}+\beta_{3}^{r}\cdot \log\left(p_{n}\right)+\beta_{4}^{r}\cdot \log\left(1-p_{n}\right)+c_{n}^{r}, \end{array}$$

where *k* denotes the testing block index, *n* the trial index, and *β* ^*r*^ the regression coefficients. The first coefficient $\beta_{0,k}^{r}$ is a fixed intercept grouped by testing condition *k* (i.e., generative probabilities of the sequences). Then, for each participant, at each channel and at each time point from −0.5 to 1 s around stimulus onset, the EEG signal *z*_*n*_ was regressed on the Bayesian prediction error (BPE) *e*_*n*_ and residual confidence $c_{n}^{r}$ (omitting the dependence of the regressors upon the model *M*_*i*_ and its parameter *ω*_*i*_ for clarity):

[15] $$\begin{array}{c} z_{n}=\beta_{0,k}+\beta_{1}\cdot e_{n}+\beta_{2}\cdot c_{n}^{r}+\epsilon . \end{array}$$

The regressions were computed across all available trials.

The two considered regressors—BPE and residual confidence—deduced from the optimal inference were not linearly related, enabling to compute and safely interpret the regression coefficients. To confirm that they are not collinear, we computed the variance inflation factors (VIFs) for (residual) confidence against BPE (25): $\;\text{VIF}=\frac{1}{1-R^{2}}$ , where *R* ^2^ is the coefficient of determination obtained when linearly regressing (residual) confidence on BPE. Unless stated otherwise, “residual” is assumed when mentioning confidence in this work. Significance of the regression coefficients across participants was assessed using one-sample t -tests against 0. The significance level was set to 0.05 and corrected for multiple comparisons across time points and selected electrodes (C3, Cz, FCz, CPz, and C4) with the false discovery rate (FDR) correction.

As suggested by a reviewer, we also assessed the effects of BPE and confidence on the EEG responses from all the electrodes, using cluster-based significance tests (by shuffling the regressors across trials). With this approach, *SI Appendix*, Figs. S8 and S9 show all the significant clusters that are found for the TP and IF models, respectively, ordered in decreasing order of cluster-level significance. It can be noted that the largest significant clusters are concentrated around: 1) the N2-P2 components for confidence and 2) later potentials for prediction errors, both effects being centrally distributed around the vertex. These analyses provide additional validations of our main results.

## Supplementary Material

Appendix 01 (PDF)

## Data Availability

The behavioral and EEG data sets are publicly available on the OSF repository at https://osf.io/8xvtg/ (DOI https://doi.org/10.17605/OSF.IO/8XVTG). The codes used to conduct the experiments, generate the model outcomes, analyze the data and produce all figures are openly available at https://doi.org/10.5281/zenodo.7509927.
