# Supplementary material for: Confidence of probabilistic predictions modulates the cortical response to pain
Source: Proc Natl Acad Sci U S A. 2023 Jan 20;120(4):e2212252120. doi: 10.1073/pnas.2212252120 (PMC9942789; doi:10.1073/pnas.2212252120)
Supplement: Supplementary file 1 — Appendix 01 (PDF) [file pnas.2212252120.sapp.pdf]

Supporting Information for

Confidence of probabilistic predictions modulates the cortical response to pain

Dounia Mulders, Ben Seymour, André Mouraux, Flavia Mancini

Corresponding authors: Dounia Mulders, Flavia Mancini.
E-mails: dmulders@mit.edu, flavia.mancini@eng.cam.ac.uk

This PDF file includes:

Supporting text
Figs. S1 to S9
Table S1
SI References

Supporting Information Text

Participants and task

Thrity-six participants were recruited for this study. As described in the manuscript, 5 subjects were excluded from the analyses because 2 of them made 1-2 errors in the pre- or postcheck sessions (most likely due to distraction), 2 of them fell asleep during the task and the task had different parameters for 1 pilot participant. Table S1 indicates, for all participants, the temperatures employed throughout the experiment (stimuli of intensities I_1 and I_2), the outcomes of the check stimulus discrimination sessions and the exclusion reasons.

During the experiments, all participants received 10 sequences of 100 stimuli from two intensities I_1 (cool) and I_2 (warm). Each sequence was generated according to a Markovian process. As illustrated in Fig. S1, similar numbers of stimuli from both intensities were delivered to all participants (a), and they all predicted similar numbers of transitions from both intensities (b).

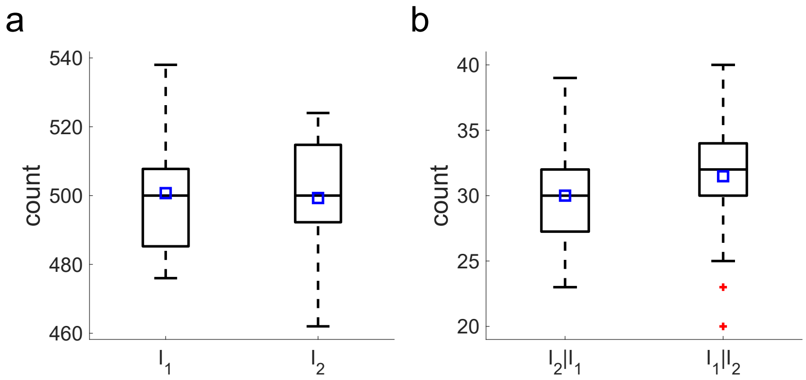

Fig. S1. The number of stimuli from both intensities and the number of rated transitions are balanced.
a, Numbers of stimuli from each intensity delivered to the participants along all the testing blocks. Participants ($N = 31$) received balanced numbers of stimuli from both intensities (mean difference between the numbers of I_1 and I_2: -1.419, $t_{30} = -0.25$, $p = 0.804$, Cohen's $d = -0.045$). Blue square: mean number of stimuli.
b, Likewise, participants rated, on average, similar numbers of both types of transitions (mean difference of numbers of rated transitions from I_1 and from I_2: 1.484, $t_{30} = 1.054$, $p = 0.3$, Cohen's $d < 10^{-5}$).

EEG modulation using alternative inference models

In order to identify how inferring the sequence statistics modulates the EEG responses, these signals can be regressed on the confidence and prediction error (PE) obtained trial-by-trial from a learning model. In the manuscript, this is done using the model which best approximates the participants' probability estimates, i.e. a Bayesian model learning the sequence TPs with a time constant of 8 stimuli (Figs. 5 and 6). This analysis reveals a strong negative association between the vertex potential (VP) and confidence. Hereunder, we show that these modulations are preserved if other plausible models are employed to derive the confidence and PE.

First, if the raw confidence is used as regressor instead of the residual confidence (as defined in (14)), the main results remain unchanged (Fig. S2).

Likewise, Fig. S3 illustrates similar findings if the 'preferred' learning model of each participant (based on the fitting of the behavioral reports) is considered to obtain the regressors. Because only a minority of participants preferred to learn the IF or AF rather than the TPs, it does not significantly affect the outcomes shown in Figs. 5 and 6.

Finally, if the second model which best approximates the overall participants' behavior is considered, i.e. a Bayesian model learning the sequence IF with a time constant of 8 stimuli, Fig. S4 shows that the correlation between the VP and confidence is preserved (b), and that the PE modulates late EEG responses more strongly (c).

Table S1. Outcomes of the pre- and post-check sessions. For each participant recruited, number of mistakenly identified stimuli during the pre- and post-check sessions, over a total of 15 stimuli, temperatures of the 2 types of stimuli during the experiment and age of the participants. Rows highlighted in red indicate participants who were excluded from the analyses.

	mistakes (#/15)					
ID	precheck	postcheck	I_1 (°C)	I_2 (°C)	age	exclusion
1	0	0	15	51	21	different stimulus durations used (500ms)
2	0	0	15	58	22	
3	0	0	15	58	28	
4	0	0	15	58	20	
5	0	0	15	58	22	
6	0	0	15	57	22	
7	0	0	15	58	24	
8	0	0	15	58	21	
9	0	0	15	58	27	
10	0	0	15	58	20	
11	1	/	20	58	25	1 precheck mistake
12	0	0	15	58	23	
13	0	0	15	58	22	
14	0	0	15	58	27	
15	0	/	15	58	39	participant fell asleep
16	0	0	15	58	22	
17	0	0	15	57	23	
18	0	0	15	58	20	
19	0	0	15	57	24	
20	0	0	15	57	26	
21	0	0	15	58	26	
22	0	0	15	58	18	
23	0	0	15	58	20	
24	0	0	15	58	28	
25	0	0	15	57	30	
26	0	0	15	57	25	
27	0	0	15	57	26	
28	0	2	15	57	24	2 postcheck mistakes
29	0	0	20	58	25	
30	0	0	15	58	23	
31	0	0	20	58	18	
32	0	0	15	57	21	
33	0	/	15	57	52	participant fell asleep
34	0	0	15	57	27	
35	0	0	20	57	24	
36	0	0	20	57	18	

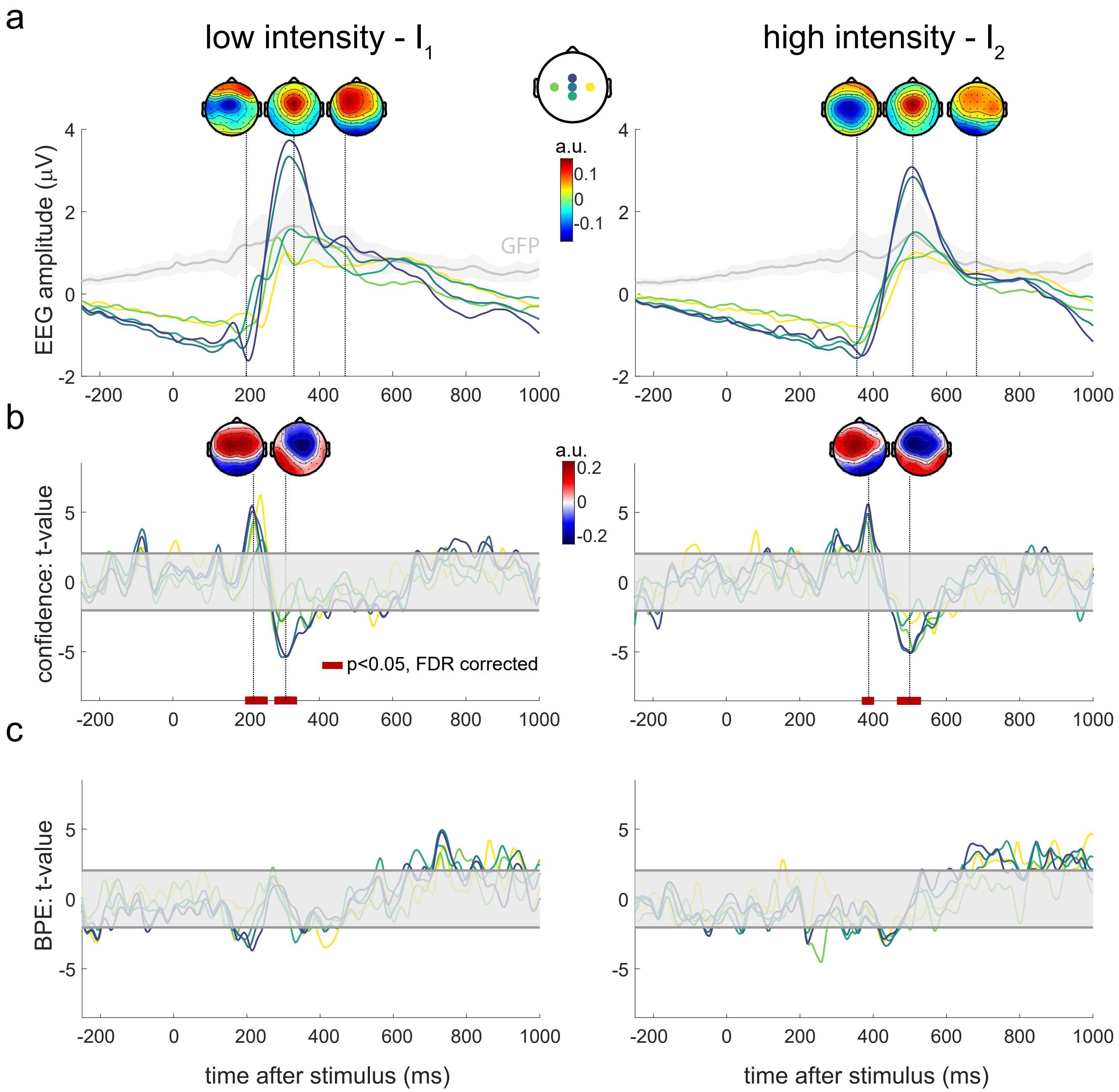

Fig. S2. EEG modulation by the Bayesian prediction error (BPE) and raw confidence. Similar to Fig. 5 but using the raw instead of residual confidence as regressor.
$\mathbf{a}$, EEG responses averaged over trials, blocks and participants, for low (left) and high (right) stimulation intensities. Global Field Power (GFP) time courses are shown in gray, with shaded SD across participants. Labels of depicted electrodes, whose positions are shown in the topoplot at the center: C3, Cz, FCz, CPz, C4.
$\mathbf{b}$, Encoding of raw confidence in the EEG responses – t-statistics for the regression coefficients associated with the model confidence.
$\mathbf{c}$, Encoding of BPE in the EEG responses – t-statistics for the regression coefficients associated with the BPE.
In **b** and **c**, confidence and BPE are obtained from the model which best explains the participants' behavior: a Bayesian model learning the TPs with an integration time constant of 8 stimuli. The shaded horizontal areas centered around 0 indicate the non-significant regions for $p < 0.05$, two-tailed. Red bars at the bottom of the plots show intervals where the regression coefficients are significantly different from 0 after False Discovery Rate (FDR) correction of the significance levels. BPE and raw confidence are not collinear: the average variance inflation factors (VIFs) of raw confidence against BPE $=$ 1.1 and 1.08 for I_1 and I_2 respectively, far below 5 (1) ($R^2 = 8.92$ and 7.36% when we regress the raw confidence on BPE).

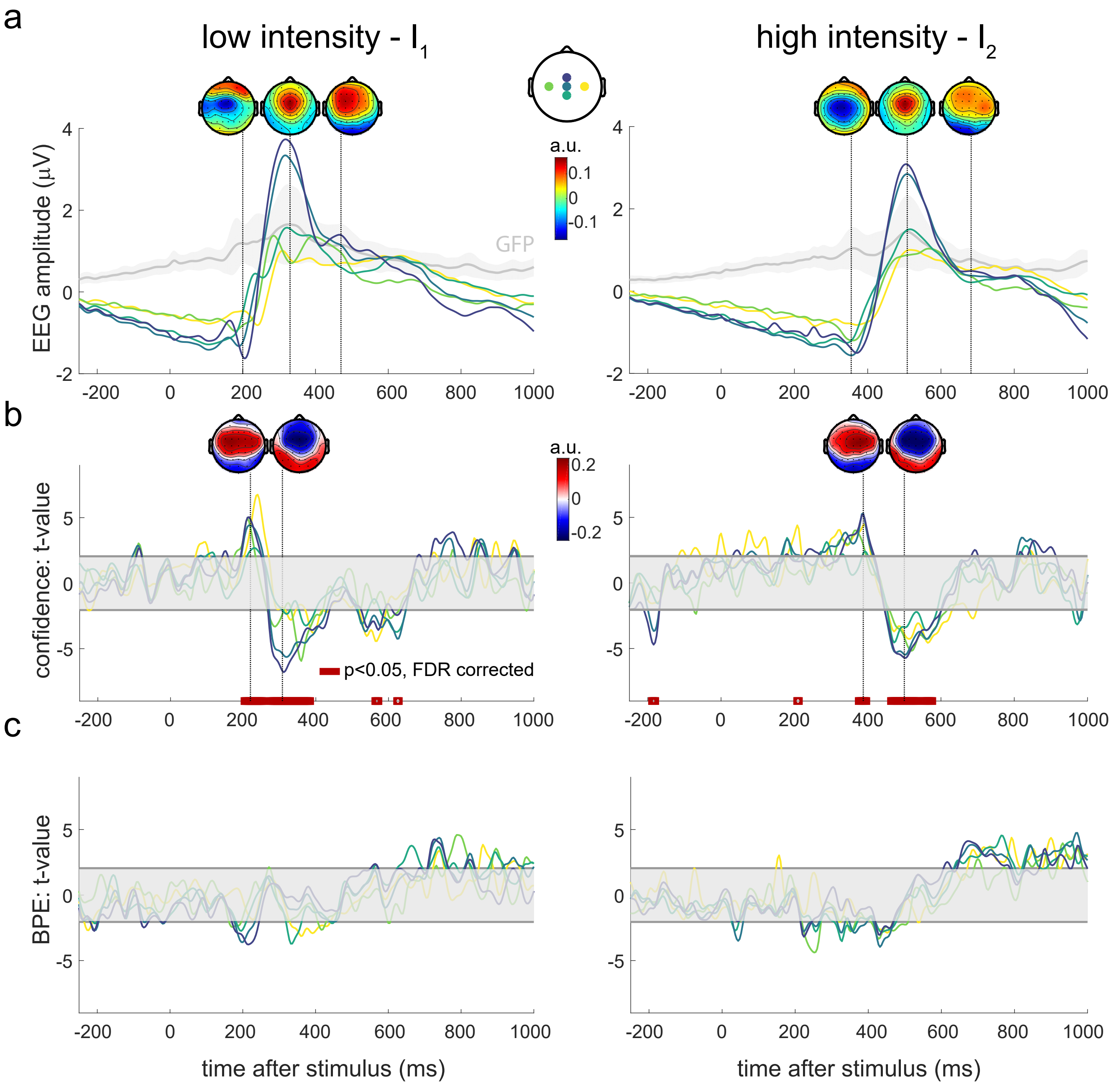

Fig. S3. EEG modulation by the Bayesian prediction error (BPE) and residual confidence. Similar to Fig. 5 but using the model which best explains each participant's behavior (Bayesian model learning the IF, AF or TPs, with an integration time constant of 8 stimuli) to compute the confidence and prediction errors.

a, EEG responses averaged over trials, blocks and participants, for cool (left) and warm (right) stimuli. Global Field Power (GFP) time courses are shown in gray, with shaded SD across participants. Labels of depicted electrodes, whose positions are shown in the topoplot at the center: C3, Cz, FCz, CPz, C4.

b, Encoding of confidence in the EEG responses – t-statistics for the regression coefficients associated with the model confidence.

c, Encoding of BPE in the EEG responses – t-statistics for the regression coefficients associated with the BPE.

The shaded horizontal areas centered around 0 indicate the non-significant regions for $p < 0.05$, two-tailed. Red bars at the bottom of the plots show intervals where the regression coefficients are significantly different from 0 after False Discovery Rate (FDR) correction of the significance levels.

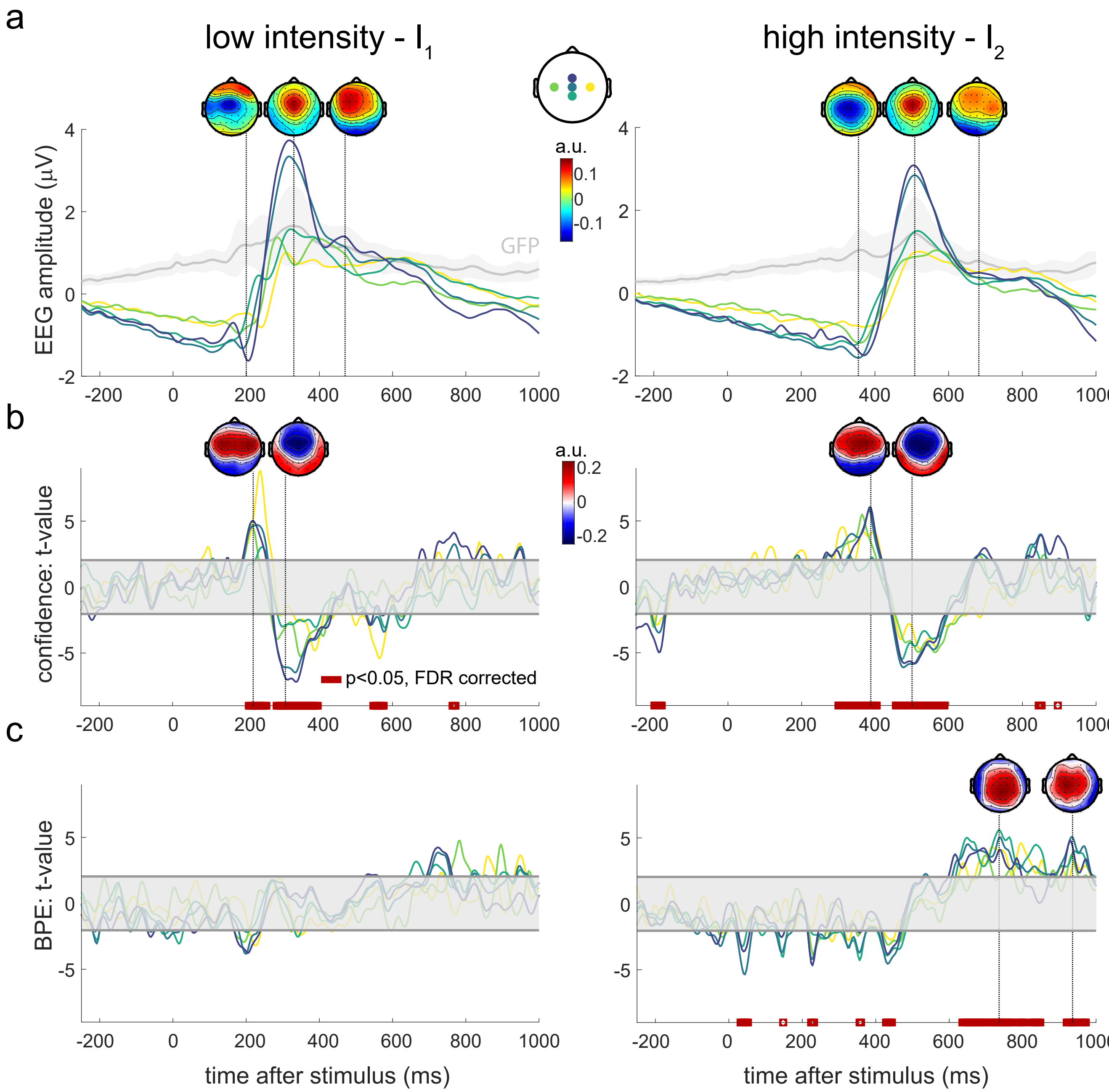

Fig. S4. EEG modulation by the Bayesian prediction error (BPE) and residual confidence. Similar to Fig. 5 but using a Bayesian model learning the IF instead of TPs. **a**, EEG responses averaged over trials, blocks and participants, for low (left) and high (right) stimulation intensities. Global Field Power (GFP) time courses are shown in gray, with shaded SD across participants. Labels of depicted electrodes, whose positions are shown in the topoplot at the center: C3, Cz, FCz, CPz, C4.
b, Encoding of residual confidence in the EEG responses – t-statistics for the regression coefficients associated with the model confidence.
c, Encoding of BPE in the EEG responses – t-statistics for the regression coefficients associated with the BPE.
In **b** and **c**, confidence and BPE are obtained from the second model which best explains the participants' behavior: a Bayesian model learning the IF with an integration time constant of 8 stimuli. The shaded horizontal areas centered around 0 indicate the non-significant regions for $p < 0.05$, two-tailed. Red bars at the bottom of the plots show intervals where the regression coefficients are significantly different from 0 after False Discovery Rate (FDR) correction of the significance levels. Again, BPE and confidence are not collinear: the average VIFs of confidence against BPE $=$ 1.0048 and 1.0058 for I_1 and I_2 respectively, far below 5 (1) ($R^2 = 0.47$ and 0.58% when we regress confidence on BPE).

Bayesian model updates

In Bayesian models, a sequence transition probability (TP) is continuously estimated through the computation of the posterior distribution of this TP given the past stimuli $y_{1:n}$ (see equations [1]-[2]). Before receiving stimulus y_{n+1}, a prediction about the forthcoming intensity is hence available and corresponds to the mean of the posterior distribution given $y_{1:n}$ (see [6]). Examples of posterior distributions and their mean before (gray) and after (black) receiving a stimulus within a sequence are shown in Fig. S5.

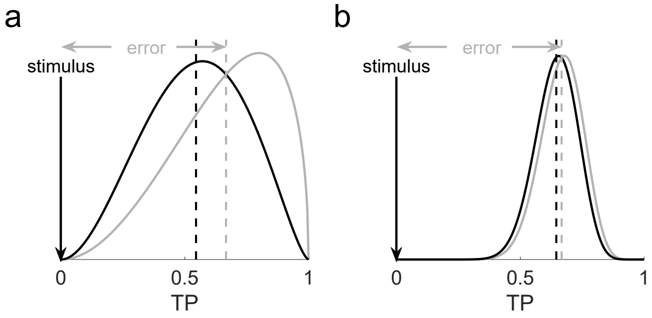

Fig. S5. Stimulus probabilities before and after receiving a stimulus in the Bayesian model. The transition probability (TP) between stimulus intensities is continuously estimated along the sequence. In a Bayesian framework, a TP is estimated based on past stimuli using a Beta distribution. The distribution before receiving a stimulus (gray, with dashed mean) is updated (black, with dashed mean) according to the last stimulus received. In **a** and **b**, the prediction errors are the same.
a, When confidence (precision) is low, the update induced by one observation is large.
b, When confidence (precision) is high, the update induced by one observation is smaller.

Parameter and model recovery analyses

In order to assess the robustness of our model and parameter selection procedures, we conducted parameter and model recovery analyses. The idea is to generate data using the true models and to determine to which extent the selection procedures can recover the true models and their parameters (2, 3). Here, with the Bayesian models (learning AF, IF or TPs), the parameters are the integration time constant. Because the model predictions (i.e. probability estimates) are deterministic for a given sequence, data were simulated by sampling probability estimates from the Beta distribution estimated at each time step (see [3]-[4]-[5]). Results of the parameter and model recovery analyses can be found in Fig. S6. For all three models, Fig. S6a shows the recovered integration time constants (y-axis) for 30 simulations using all $N = 31$ parameters fitted to the participants' reports (true parameters, x-axis). Then, Fig. S6b illustrates that the three models themselves can be reliably re-identified when fitting noisy data.

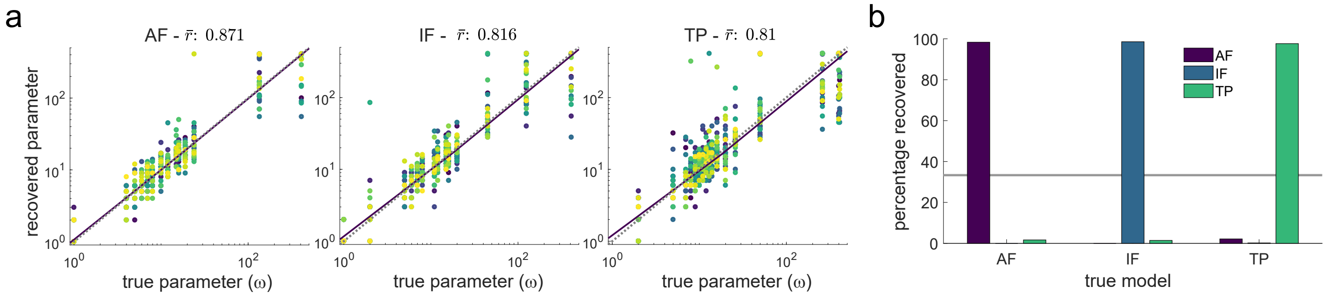

Fig. S6. Parameter and model recovery for the Bayesian learner. To assess whether the three Bayesian models and their hyper-parameter (the integration time constant ω) are identifiable, we simulated data using each model with different parameters and fitted the models to these synthetic data like we did it to the behavioral reports. Since the model predictions (i.e. probability estimates) are deterministic for a given sequence, data were simulated by sampling probability estimates from the Beta distribution estimated at each time step (see Methods). For each model (learning AF, IF or TPs), we consider all the optimal time constants that were fitted to the individual behavioral data (2). Using each time constant and model, 30 synthetic data sets were built based on the same number of sequences and probability estimates as for the real participants (10 sequences were generated with the TPs indicated in Fig. 1d and probability estimates were sampled every 15±3 stimuli).
a, The parameter recovery analysis indicates that the integration time constant can be reliably recovered despite readout noise for all three models in our experiments (Pearson correlation coefficient between true and fitted $\omega = 0.871, 0.816, 0.81$). Scatter plots of fitted vs. true parameters, with one color per simulation ($n = 30$). Dotted line: identity, thick plain black line: linear fit.
b, The model recovery shows that the three models are highly identifiable in our experimental setting, with 98.3, 98.6 and 97.6% of correctly recovered models for the model learning AF, IF and TPs respectively.

Gamma-band oscillations (GBOs) induced by the stimuli

Using the epochs before applying the low-pass filter described in the Materials and Methods section of the article, we checked whether the hot stimuli induced more GBOs than the cool ones, a typical EEG correlate of pain perception (4, 5).

To this aim, we first computed short-time Fourier transforms (STFTs) of the amplitudes of each trial for each participant at FCz, from 40 to 140 Hz in steps of 2 Hz and from -1 to 1.5 seconds after stimulus onset. We considered 100ms width Gaussian windows for the STFTs to reach a reasonable time resolution for GBOs. We then averaged the single-trial STFTs per participant and computed the percentage of stimulus-induced changes of amplitude as (5, 6): $E_R(t,f) = 100 \cdot \frac{S(t,f)-R(f)}{R(f)}$, where $S(t,f)$ is the signal amplitude at time t and frequency f, $R(f)$ is the average baseline amplitude at frequency f, obtained by averaging the amplitude in the $[-0.5,-0.1]$ seconds time window, and E_R is expressed in %.

Although we could not reliably detect GBOs in all participants (as it is commonly the case), the hot stimuli induced significantly more GBOs than the cool ones (in the interval $[60,90]$ Hz - $[0.15, 1.5]$ s for I_2 vs. I_1: mean difference of 0.81%, $t_{30} = 2.46$, $p = 0.02$, Cohen's $d = 0.44$). The grand averaged STFTs are displayed in Fig. S7. These maps suggest that the larger GBOs in response to warm I_2 stimuli mostly occurred in late time windows. Following previous works (5, 7, 8) suggesting that pain-induced GBOs can occur concomitantly with the VPs, we also compared the amplitudes of GBOs induced by the cool and hot stimuli along the time intervals defined by their respective grand mean N2 waves, i.e. $[170, 240]$ms for I_1 and $[320, 430]$ms for I_2. Along these time intervals, there was no significant difference of induced GBO amplitudes in the $[60, 90]$ Hz range: mean difference of 0.11%, $t_{30} = 0.26$, $p = 0.8$, Cohen's $d = 0.05$.

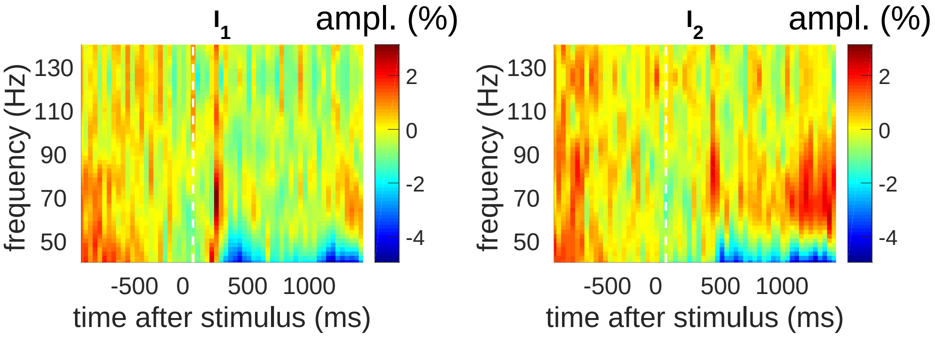

Fig. S7. Grand average time-frequency maps of the stimulus-induced changes of amplitude (%) at FCz. In the post-stimulus interval, there is a late preferential enhancement of amplitudes in the $[60, 80]$ Hz range in response to I_2 (warm, right) stimuli compared to I_1 (cool, left). Short-time Fourier transforms (STFTs) with 100ms width Gaussian windows were used.

EEG modulation by the inference over all electrodes

In the manuscript, we reported how confidence and BPE modulate the VP (by focusing on central electrodes). Here, we also assessed the effects of these inferential parameters on the EEG responses from all the electrodes, using cluster-based significance tests (by shuffling the regressors across trials). Results are displayed in Figs. S8 and S9 for the Bayesian TP and IF models respectively, with one sub-plot for each significant cluster found, ordered in decreasing order of cluster-level significance. These analyses validate our main findings: using both models, the largest significant clusters are concentrated around (1) the N2-P2 components for confidence and (2) later potentials for prediction errors, both effects being centrally distributed around the vertex.

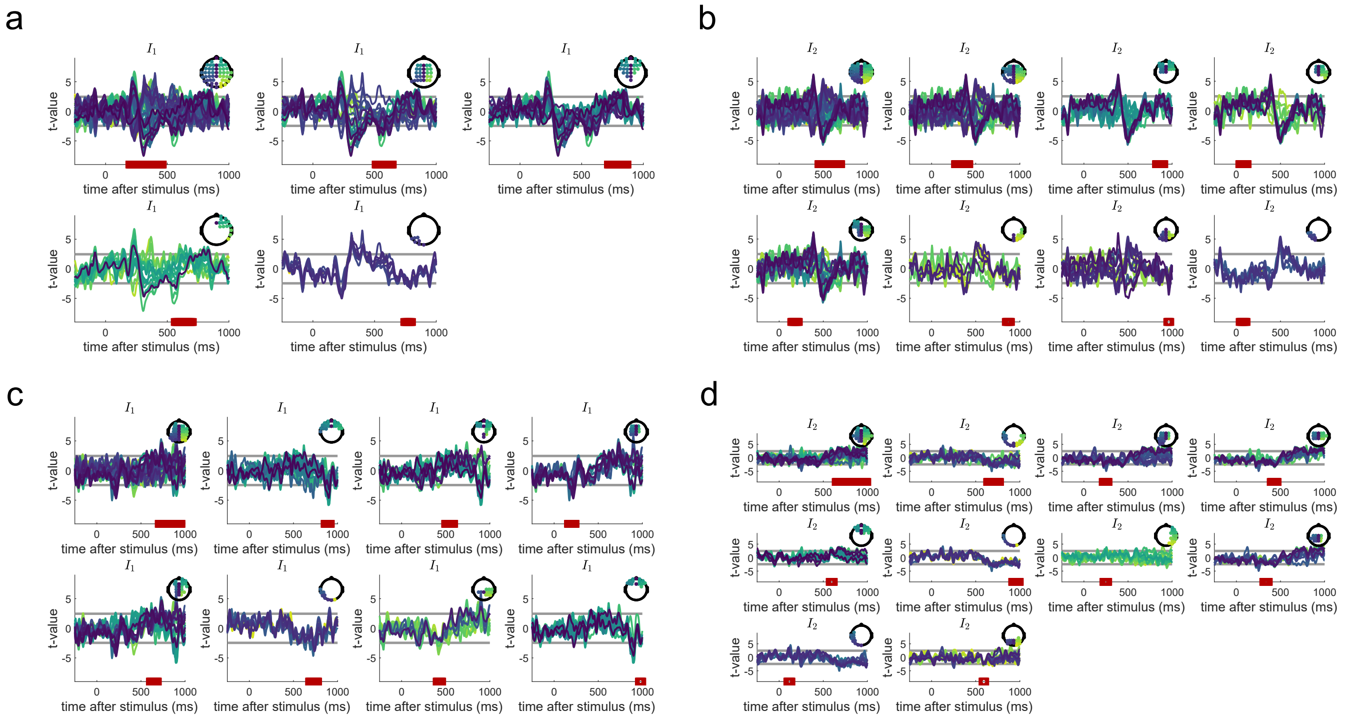

Fig. S8. EEG modulation by the Bayesian prediction error (BPE) and residual confidence.. Using the Bayesian model learning the TPs with a time constant of 8 stimuli. Each color is associated with one electrode as indicated in the topoplots. Red bars at the bottom of the figures indicate time clusters along which there are significant effects at the represented channels (cluster-forming $p<0.05$ two-tailed and cluster-level $p<0.01$, using 2000 permutations of the regressors across trials).
$\mathbf{a}$, t-statistics for the regression coefficients associated with the confidence, I_1 stimuli.
$\mathbf{b}$, t-statistics for the regression coefficients associated with the confidence, I_2 stimuli.
$\mathbf{c}$, t-statistics for the regression coefficients associated with the prediction error, I_1 stimuli.
$\mathbf{d}$, t-statistics for the regression coefficients associated with the prediction error, I_2 stimuli.

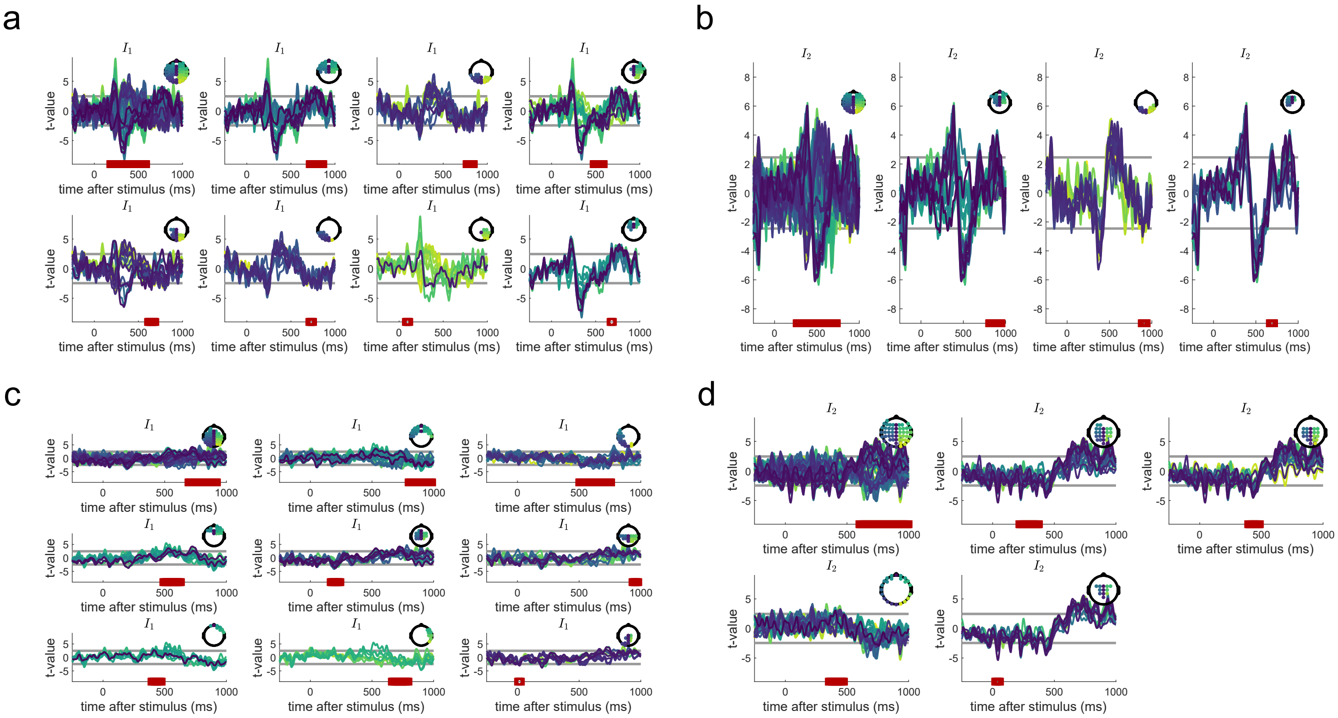

Fig. S9. EEG modulation by the Bayesian prediction error (BPE) and residual confidence.. Using the Bayesian model learning the IF with a time constant of 8 stimuli. Each color is associated with one electrode as indicated in the topoplots. Red bars at the bottom of the figures indicate time clusters along which there are significant effects at the represented channels (cluster-forming p<0.05 two-tailed and cluster-level p<0.01, using 2000 permutations of the regressors across trials).
a, t-statistics for the regression coefficients associated with the confidence, I_1 stimuli.
b, t-statistics for the regression coefficients associated with the confidence, I_2 stimuli.
c, t-statistics for the regression coefficients associated with the prediction error, I_1 stimuli.
d, t-statistics for the regression coefficients associated with the prediction error, I_2 stimuli.

References

1. S Sheather, *A modern approach to regression with R.* (Springer Science & Business Media), (2009).
2. JB Heald, M Lengyel, DM Wolpert, Contextual inference underlies the learning of sensorimotor repertoires. *Nature* **600**, 489–493 (2021).
3. RC Wilson, AG Collins, Ten simple rules for the computational modeling of behavioral data. *Elife* **8**, e49547 (2019).
4. M Ploner, C Sorg, J Gross, Brain rhythms of pain. *Trends Cogn. Sci.* **21**, 100–110 (2016).
5. G Liberati, et al., Gamma-band oscillations preferential for nociception can be recorded in the human insula. *Cereb. cortex* **28**, 3650–3664 (2018).
6. G Pfurtscheller, FL Da Silva, Event-related EEG/MEG synchronization and desynchronization: basic principles. *Clin. neurophysiology* **110**, 1842–1857 (1999).
7. J Gross, A Schnitzler, L Timmermann, M Ploner, Gamma oscillations in human primary somatosensory cortex reflect pain perception. *PLoS biology* **5**, e133 (2007).
8. Z Zhang, L Hu, YS Hung, A Mouraux, G Iannetti, Gamma-band oscillations in the primary somatosensory cortex — a direct and obligatory correlate of subjective pain intensity. *J. Neurosci.* **32**, 7429–7438 (2012).
